# The Dry Season in Haiti: a Window of Opportunity to Eliminate Cholera

**DOI:** 10.1371/currents.outbreaks.2193a0ec4401d9526203af12e5024ddc

**Published:** 2013-06-10

**Authors:** Stanislas Rebaudet, Pierre Gazin, Robert Barrais, Sandra Moore, Emmanuel Rossignol, Nickolson Barthelemy, Jean Gaudart, Jacques Boncy, Roc Magloire, Renaud Piarroux

**Affiliations:** Aix-Marseille University, UMD 3, Marseilles, France; Aix-Marseille University, UMD 3, Marseilles, FranceIRD; Ministère de la Santé Publique et de la Population, Port-au-Prince, Haiti; Aix-Marseille University, UMD 3, Marseilles, France; Ministère de la Santé Publique et de la Population, Port-au-Prince, Haiti; Ministère de la Santé Publique et de la Population, Port-au-Prince, Haiti; Aix-Marseille University; Ministère de la Santé Publique et de la Population, Port-au-Prince, HaitiLNSP, Port-au-Prince; Ministère de la Santé Publique et de la Population, Port-au-Prince, Haiti; Aix-Marseille University, UMD 3, Marseilles, France

## Abstract

Background: Since the beginning of the cholera epidemic in Haiti, attack rates have varied drastically with alternating peak and lull phases, which were partly associated with the fluctuating dry, rainy and cyclonic seasons. According to a study conducted in 2012, the toxigenic V. cholerae O1 strain responsible for the outbreak did not settle at a significant level in the Haitian aquatic environment. Therefore, we hypothesize that some areas of lingering cholera transmission during the dry season could play an important role in the re-emergence of outbreaks during the rainy season. Our objective was therefore to describe the dynamics of cholera and assess the fight against the disease during the dry season. Methods: A field study was conducted from February 19 to March 29, 2013. After identifying the affected communes by analyzing the national cholera database, we visited corresponding health facilities to identify patient origins. We then conducted a field assessment of these foci to confirm the presence of cholera, assess factors associated with transmission and examine the activities implemented to control the epidemic since the beginning of the current dry season. Results: We found that the great majority of Haitian communes (109/140) presented no sign of cholera transmission in February and March 2013. Suspected cases were concentrated in a small number of urban and rural areas, almost all of which were located in the northern half of the country and often in inland locales. In these areas, community health activities appeared insufficient and were often inappropriately targeted. Out of 49 analyzed foci, only 10 had benefited from at least one intervention involving the distribution of water treatment products together with an awareness campaign since December 2012. Conclusion: Cholera continues to affect Haiti as observed in early 2013; however, activities implemented to interrupt cholera transmission appear insufficient and poorly suited. This deficiency in the fight against cholera, especially at a period when transmission is weak, may explain the persistence of cholera even in the absence of significant aquatic reservoirs in Haiti.

## Introduction

In October 2010, the importation of a toxigenic *Vibrio cholerae* strain in Haiti 1 2 3 led to a massive epidemic that rapidly spread throughout all 140 Haitian communes 4 5. By the end of March 2013, the Public Health and Population Ministry (MSPP) had reported 652,730 cases, corresponding to an attack rate of 6.4%. With 8,060 reported deaths, the global case fatality rate was 1.2%. This historic epidemic, which occurred in a small but densely populated country (10.2 million inhabitants in 27,750 km2), accounted for over half of cholera cases and over one-third of cholera-associated deaths reported globally between 2010 and 2011 6 . The disease also spread throughout the rest of Hispaniola island, and by late 2012, the Dominican Republic had recorded 29,433 cases and 422 deaths associated with cholera 7.

Since the beginning of the epidemic, cholera incidence in Haiti has been characterized by alternating peak and lull phases, which were partly associated with the fluctuating dry, rainy and cyclonic seasons 1 (Figure 1).

**Evolution of the daily suspected cholera cases and rainfall between September 2010 and March 2013.**
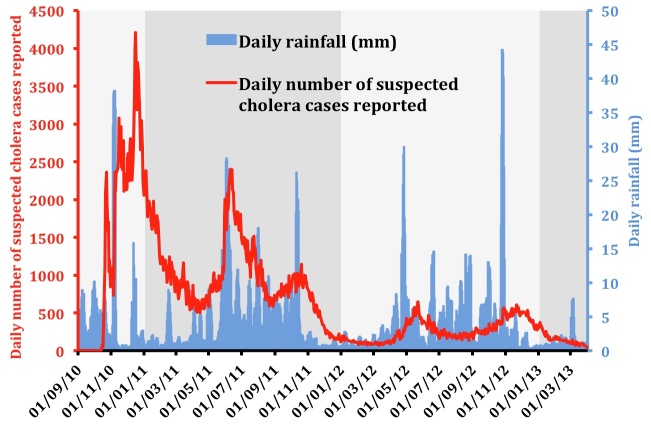
Accumulated rainfall data were obtained from satellite estimates (TMPA-RT 3B42RT derived), averaged on the position 18.25N-19.75N / 74.25W-71.75W, and available at: http://disc2.nascom.nasa.gov/Giovanni/tovas/realtime.3B42RT_daily.2.shtml.

Haiti experienced the first marked decrease in cholera transmission during the dry season in early 2012 (67 cases/day reported in March). At that time, cholera had almost completely disappeared from the Southern Peninsula as well as the North-East and North-West departments according to the Haitian National Cholera Surveillance System 8. In contrast, residual transmission was recorded in a few dozen communes located in the North (DSN), Artibonite (DSA) and Centre (DSC) departments as well as the conurbation of Port-au-Prince (West department). During the rainy season of April-May 2012, the reappearance of the epidemic was first reported in these geographic areas, before spreading to the areas where transmission seemed to have stopped during the dry season. By the end of 2012, 903 additional cholera-associated deaths had been reported.

However, the evolution of cholera epidemics is always difficult to predict, and there is no undisputable study demonstrating that cholera will settle in Haiti for decades to come. Despite poor access to improved drinking water sources (69% of the population in 2010) and very poor use of improved sanitation facilities (17% of the population in 2010)^9^, the country and the rest of the island of Hispaniola had been spared from cholera for at least a century and probably since the beginning of recorded history 10. Moreover, a Franco-Haitian study conducted in 2012 (Baron S et al., in process) has shown that the O1 strain responsible for the outbreak was overwhelmed by local non-O1 V. cholerae strains in a large set of Haitian aquatic environments. None of the rare O1 strains isolated in this study proved to be toxigenic. Thus, there is currently no evidence of a perennial and significant environmental reservoir for toxigenic *V. cholerae* in Haiti.

Therefore, we hypothesize that some areas of lingering cholera transmission during the dry season play an important role in the persistence of epidemics from one rainy season to the next and that the struggle against cholera in Haiti has failed to take advantage of the dry season opportunity. In this study, we aimed to describe both the dynamics of cholera during the dry season and the fight against the disease by targeting the remaining foci.

## Methods

At the request of the MSPP, a field study was conducted from February 19 to March 29, 2013 to both establish an inventory of the remaining cholera transmission foci during the dry season and assess the prevention actions carried out by Haitian and international organizations. The four components of the survey consisted of the following aspects: (1) identify affected communes by analyzing the information available in the national database; (2) identify transmission foci based on the basic data recorded by cholera treatment health structures; (3) conduct a field study of the principal foci to confirm the presence of cholera, assess factors associated with transmission and examine the actions taken to control the epidemic since the beginning of the current dry season (in collaboration with representatives of the National Department of Drinking Water and Sanitation (DINEPA)); and (4) perform interventions at the household level involving public awareness campaigns and the distribution of chlorine tablets.

Cholera-associated morbidity and mortality data at the commune level were provided by the Haitian Directorate of Epidemiology Laboratory and Research (DELR), which gathers, validates and analyzes anonymous data that are prospectively collected in the field by epidemiological surveillance officers. According to the WHO standard definition 11, a probable cholera case is defined as a patient aged 5 years or older who develops acute watery diarrhea, with or without vomiting, located in an area where there is a cholera epidemic. In Haiti, all acute watery diarrhea cases are reported as suspected cholera, but the surveillance system separately records cases <5 and ≥5 years-old. Bacteriological confirmation of cases is routinely performed at the National Laboratory of Public Health (LNSP) using standard methods 12.

The identification of concentrations of active cholera transmission was carried out by analyzing February and March 2013 cholera national databases. We assessed any commune presenting a bacteriological-confirmed cholera case OR a suspected cholera death (associated with concomitant reported cholera cases) OR more than 1 suspected cholera case per day (excluding patients less than 5 years of age to enhance the case definition specificity). We then visited the health facilities of as many at-risk communes as possible to identify the exact origin of cholera patients by interviewing the medical staff and reviewing the case registers of the last 1 to 3 months.

Field investigations were subsequently performed in identified suspected cholera foci to search for possible factors linked to cholera transmission. People and local health actors were also interviewed regarding the actions undertaken to stop the spread of cholera since the beginning of the dry season in December 2012. In Port-au-Prince, data on patient origin was provided by the staff of Médecins sans Frontières – Holland, who also described the actions performed to limit cholera transmission. Due to the time limitation, not all identified at-risk communes and transmission foci were investigated. However, medical staff declaring the suspected cholera cases and/or local epidemiologists were interviewed by phone.

Maps of cholera morbidity, mortality and prevention interventions were generated using Quantum-GIS® v1.8.0 (Open Source Geospatial Foundation Project, Beaverton, OR, USA).

## Results

From December 1, 2012 to March 31, 2013, 21,695 suspected cholera cases (≥5 years of age) and 238 related deaths were reported by the national surveillance system. However, most cases occurred in December and January (16,700 cases and 208 deaths) and a strong decrease in cholera incidence was noted in February and March with only 4,995 recorded cases and 30 deaths. In parallel, the percentage of positive samples cultured at the LNSP dropped from 68% in December and January to 41% in February and March (Table 1).

**Table 1. Bacteriological confirmation of cholera at the LNSP. Evolution of the monthly number of positive and negative samples (% of total) from December 2012 to March 2013:** LNSP, National Laboratory of Public Health, Port-au-Prince pos, positive culture ; neg, negative culture

	December 2012		January 2013		February 2013		March 2013		Entire study period		
Department	pos	neg	pos	neg	pos	neg	pos	neg	pos	neg	Total
West	85 (77%)	25 (23%)	50 (57%)	38 (43%)	38 (39%)	59 (61%)	32 (51%)	31 (49%)	205 (57%)	153 (43%)	358
Artibonite	44 (70%)	19 (30%)	31 (70%)	13 (30%)	23 (56%)	18 (44%)	7 (26%)	20 (74%)	105 (60%)	70 (40%)	175
South-East	25 (66%)	13 (34%)	12 (80%)	3 (20%)	6 (38%)	10 (63%)	4 (36%)	7 (64%)	47 (59%)	33 (41%)	80
Centre	15 (75%)	5 (25%)	0 (0%)	8 (100%)	0 (NA)	0 (NA)	0 (0%)	15 (100%)	15 (35%)	28 (65%)	43
North	2 (100%)	0 (0%)	0 (0%)	2 (100%)	0 (0%)	4 (100%)	6 (86%)	1 (14%)	8 (53%)	7 (47%)	15
Total	171 (73%)	62 (27%)	93 (59%)	64 (41%)	67 (42%)	91 (58%)	49 (40%)	74 (60%)	380 (57%)	291 (43%)	671

As shown in Figure 2, cholera morbidity and mortality distribution also exhibited a high degree of spatial heterogeneity, particularly at the end of the study period, when cholera seemed to persist almost exclusively in the 3 departments of DSN, DSC and DSA. Notably, only a few communes were significantly affected, even at these sites. In the South Peninsula and Port-au-Prince, the few sporadic cases were associated with a very low fatality rate and were probably non-cholera-associated diarrhea.

**Monthly cholera attack rates and number of cholera-associated deaths in patients ≥5 years of age during the dry season 2012-2013:**
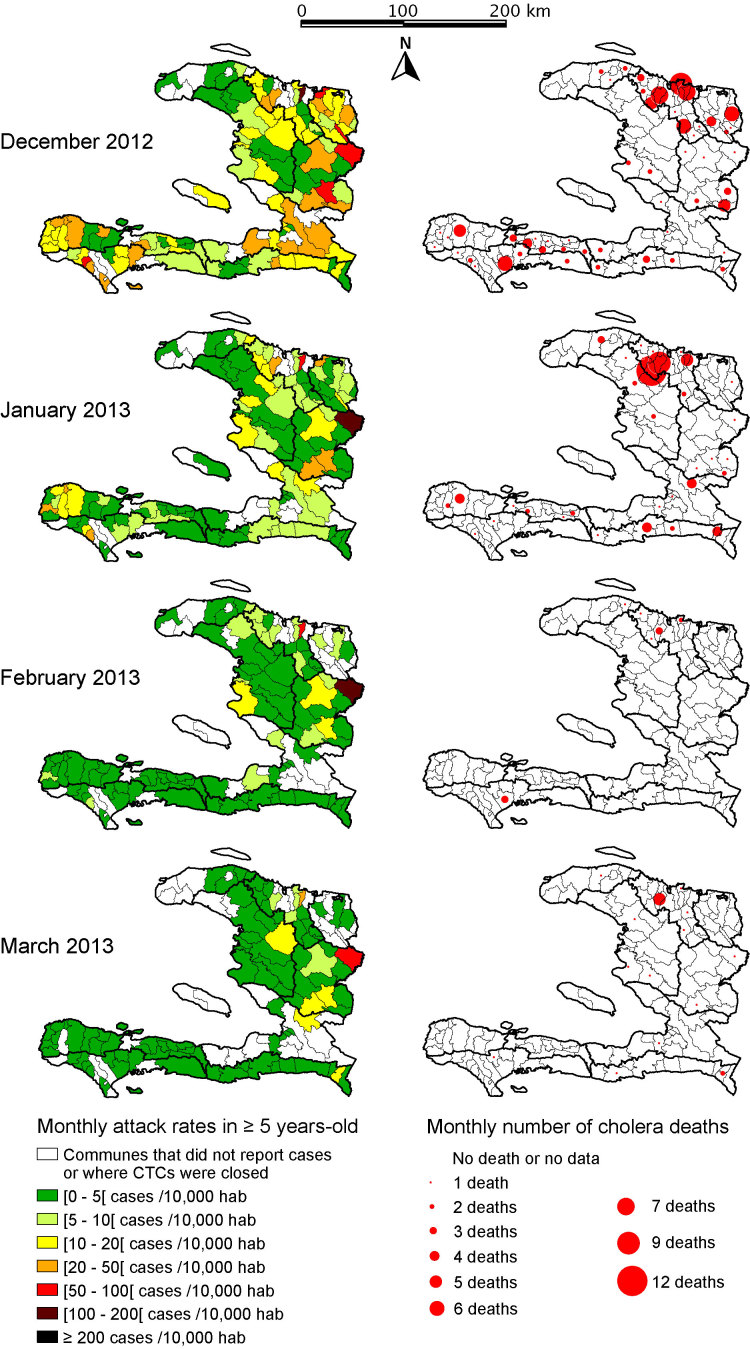

Twenty-four provincial communes with likely active cholera transmission were identified (Table 2, Figure 3). All but 8 communes were localized in the Artibonite (DSA), Centre (DSC) and North (DSN) departments. These 24 communes accounted for 69% of the total cases (≥5 years of age) recorded throughout the country, with a monthly attack rate of 6.0 cases/10,000 inhabitants. A total of 26 deaths was reported in these communes, and the cholera case fatality rate was 0.8%.

**Table 2. Communes with likely active cholera transmission in February and March 2013 (See Figure 3 for localizations):** ^1^ Port-au-Prince conurbation = communes of Port-au-Prince, Carrefour, Delmas, Petionville, Cite-Soleil, Tabarre and Kenskoff; ^2^ calculated from 2003 Census and provided by Haitian Ministry of the Interior and Territorial Collectivities; ^3^ <5 years of age and ≥5 years of age; ^4 ^cases suspected by acute watery diarrhea; ^5^ attack rates presented in cases /10,000 inhabitants.

Department	Commune	Estimated population in 2012^2^	<5^3^ No of cases^3^	≥5^3^ No of cases^4^	≥5^3^ Attack rate^5^	≥5^3^ Cholera deaths	Bacterio- logical confirmation	Date of last confirmation
Artibonite	Gonaïves	340,155	70	198	5.8	1		13/12/12
Artibonite	Gros Morne	148,627	13	141	9.5	0	Yes	14/02/13
Artibonite	Saint-Marc	254,543	60	515	20.2	1	Yes	05/03/13
Artibonite	Saint-Michel	143,679	81	198	13.8	0		
Artibonite	Verrettes	138,242	44	110	8.0	1		
Centre	Cerca-la- Source	5,397	97	113	209.4	1		
Centre	Hinche	115,381	160	223	19.3	0		
Centre	Lascahobas	43,790	60	147	33.6	0		07/12/12
Centre	Mirebalais	93,319	98	251	26.9	1		21/12/13
North	Borgne	63,885	32	55	8.6	1		
North	Cap-Haitien	261,952	43	359	13.7	3	Yes	22/03/13
North	Limbe	81,431	28	146	17.9	8		
North	Pilate	51,597	7	66	12.8	0		
North	Plaisance	66,426	13	50	7.5	1		
North	Quartier Morin	26,117	87	247	94.6	0	Yes	11/03/13
North	Saint Raphael	51,316	7	44	8.6	1		
North-West	Port de paix	194,719	13	65	3.3	1		
West-West	Saint Louis du Nord	111,070	11	60	5.4	1		
West	Cabaret	65,148	95	65	10.0	0		
West	Leogane	190,746	0	147	7.7	0		
West	Thomazeau	50,558	5	74	14.6	0		
South	Les Cayes	144,813	3	56	3.9	3		
South-East	Jacmel	178,756	2	40	2.2	0	Yes	25/02/13
South-East	Thiotte	33,341	5	54	16.2	2		
								
Total of the 24 selected provincial communes		2,855,008	1,034	3,424	12.0	26		
Port-au-Prince conurbation (7 communes)^1^		2,599,952	231	738	2.8	1	Yes	01/03/13
Rest of Haiti (109 communes)		4,913,113	301	833	1.7	3	No	
Total Haiti (140 communes)		10,368,073	1,566	4,995	4.8	30		
								

**Communes likely affected by active cholera transmission in February and March 2013 (See Table 2 for characteristics):**
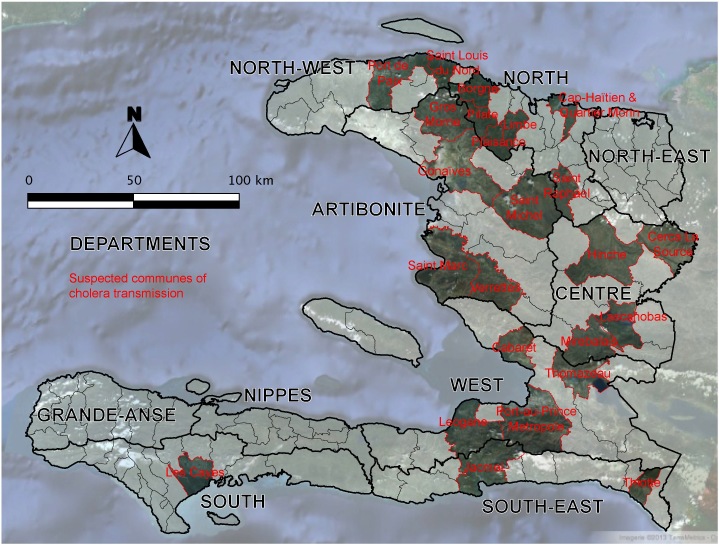
Several communes, such as Jacmel, Leogane and Plaisance, were only affected in February. Thiotte was exclusively affected in March.

A few dozen confirmed cases were reported in Port-au-Prince conurbation with only one suspected cholera-associated death (Table 1 and Table 2). However, transmission remained sporadic in the capital, thereby yielding a low attack rate (1.4 cases/month/10,000 inhabitants). Moreover, the low case fatality rate (0.1%) demonstrates that most of these cases of acute watery diarrhea were probably not due to cholera infection.

In the 109 remaining communes, the attack rate (0.8 cases/month/10,000 inhabitants) was even lower than that observed in Port-au-Prince. Only 3 deaths were recorded (1 in Camp Perrin, 1 in Grande-Rivière-du-Nord and 1 in Bainet), and cholera was not biologically confirmed for any of these cases. Therefore, in the absence of other suspected cholera cases at the same period, we considered it unlikely these cases were associated with a local transmission of cholera.

Investigations were carried out in 12 of the 24 communes with suspected active cholera transmission. These communes were located in DSN (Cap-Haïtien, Quartier-Morin, Port-Margot and Borgne), DSC (Hinche, Mirebalais and Cerca-la-Source) and DSA (Saint-Marc, Gonaïves, Saint-Michel-de-l'Attalaye, Ennery and Gros Morne). Based on the field investigations, we identified 49 areas where clusters of cases had recently been reported. Taken together, these 49 areas accounted for 56% of cases reported in these 12 communes during the analysis period.

In DSN, the principal cholera residual focus was located in the city of Cap-Haïtien. Some cases were biologically confirmed by the LNSP. Note that patients from Cap-Haïtien were also treated in the nearby commune of Quartier Morin, which remained almost free of cholera. In Cap-Haïtien, cases were concentrated in several neighborhoods without proper access to clean drinking water. The water supply network had been out of order for 25 years throughout most of the city, and the population used water treated via reverse osmosis, which does not have the anti-bacterial properties of chlorinated water. The field assessment highlighted the presence of numerous boreholes and traditional shallow wells widely contaminated with fecal matter due to their proximity to unprotected latrines. The collection of feces in plastic bags thrown onto the roof was said to be a common practice. Markets with no access to clean water and very poor food preservation practices were also likely sites of contamination.

In DSC, the commune of Cerca-la-Source reported the highest attack rates (126.0 and 83.4 cases/month/10,000 inhabitants in February and March, respectively). Only one suspected cholera-related death was reported (Figure 2). The field assessment showed that patients treated at the Cholera Treatment Center (CTC) were primarily children. As all samples delivered to LNSP for bacteriological confirmation were negative for *V. cholerae* and 90% (38/42) of the cholera rapid tests performed during the first half of March were negative, we noted a low level of cholera in this commune.

In DSA, the most active remaining foci were located in the town of Saint-Marc and several of its neighborhoods. Even though a private company sold chlorinated water, most of the population still relies on manually operated boreholes without home water treatment. In certain fishermen quarters, the general practice of defecating along the beach renders the contamination of open storage buckets of water highly likely. Cholera outbreaks occurred in several rural localities near Saint-Marc, where people rely exclusively on unprotected natural water sources. In addition to Saint-Marc, some cases were regularly reported in Gonaïves, where the municipal water network had been severely damaged by the hurricanes, as well as Saint-Michel-de-l’Attalaye. In the latter commune, most recent cases originated from the urban and peri-urban areas. Two water networks supplied this town with water and standposts. However, water was not treated for several weeks because of a chlorine shortage. In-house chlorination practices were almost never observed in those quarters, which were also largely deprived of latrines.

In all areas affected by cholera, community health activities appeared insufficient and inappropriately targeted since the beginning of the dry season 2012-2013 (Figure 4). Twenty-three of the 49 identified foci had not been previously investigated since December 2012. Thirteen had been investigated, especially in Gonaïves and Saint-Michel-de-l'Attalaye, but no intervention aimed to prevent transmission in the community had been initiated by MSPP, DINEPA or international partners. Only 13 foci among those visited had thus benefited from at least one intervention since early December 2012. At 3 of these 13 foci, prevention activities had been limited to awareness campaigns, which was sometimes due to the absence of available chlorination products (such as that found in Borgne, DSC). At least one distribution of water treatment products +/- soaps associated with an awareness campaign had nevertheless been organized in the 10 remaining foci, often with the assistance of NGOs such as Action-Contre-la-Faim (e.g., Saint-Michel-de-l'Attalaye in December) or Partners in Health (e.g., the Goyaviers section of Saint-Marc in December and January). A few distributions had been organized by the MSPP (e.g., Ennery in December and the rural Grand-Boucan portion of Mirebalais in January), while only one distribution event was launched by DINEPA in Borgne in December. Unfortunately, the majority of these distributions failed to target the principal transmission foci of the corresponding communes, which were primarily located in towns or the close outskirts, and instead exclusively focused on rural communities. Yet, some of these well-performed rural prevention events, such as that carried out in Gros Morne (DSA), proved effective with the subsequent local disappearance of cholera.

**Residual transmission foci identified in the 12 visited communes and community actions carried out since early December 2012:**
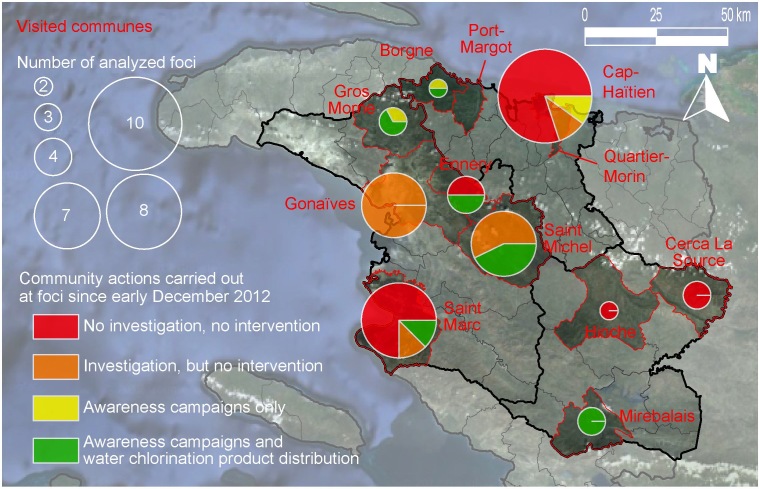

In Port-au-Prince conurbation, the low incidence and death rate associated with cholera may be due to the community actions implemented by NGOs. In particular, during the 2013 dry season, Médecins sans Frontières – Holland staff continued identifying the areas associated with clusters of cases, where they organized awareness campaigns, water treatment product distribution and free bucket chlorination stations.

## Discussion

Thirty months after cholera onset, the disease is still present in Haiti. The disease attack rate has varied considerably since the beginning of the epidemic, with peaks during the rainy season and relative lulls during the dry season in 2011 and 2012 5. Our results show that the lull was even more pronounced in February and March 2013. In particular, of the 140 Haitian communes, 109 showed no sign of cholera transmission during early 2013 for more than two months. Indeed, in these 109 communes, there has been no confirmed case, significant group of cases, or death in the context of grouped diarrheic patients. Even if small cholera outbreaks may have remained unnoticed by the surveillance system in resource-deprived areas, it is unlikely, in the current context of Haiti, that an outbreak with subsequent cholera deaths would go completely unnoticed for two consecutive months. Only sporadic suspected cholera patients were reported, with an average of less than 5 cases per commune per month, which represents less than 1 case per 10,000 inhabitants per month. It is thus likely that, in these 109 communes, almost all of isolated and unconfirmed cases were associated with afflictions other than cholera. Note that, even if these cases of diarrhea were not due to cholera, the background noise of 1 monthly case of acute watery diarrhea per 10,000 inhabitants would yield 1,000 cases per month or 12,000 cases per year for the entire country of Haiti, which could portray the false impression of persistent endemic cholera. It is therefore of upmost importance to perform a microbiological confirmation of cases as attack rates decrease.

In February and March 2013, the majority of suspected cases were concentrated in a small number of urban and rural foci, almost all of which were located in the northern half and often in inland locales. Haitian estuaries did not seem to be particularly affected. Even in these residual foci, an overestimation of cases is possible. A pooled case fatality rate of only 0.8% in the 24 communes with potential cholera transmission appears indeed implausible, considering persisting difficulties at numerous CTCs 13. A marked proportion of these cases of acute watery diarrhea, especially in children under 5 years of age, were probably not due to cholera. For instance, in the commune of Cerca-la-Source (DSC), biological tests performed on various inpatient samples were negative.

Nevertheless, cholera did not completely disappear, as active foci with laboratory-confirmed cases remained, such as that observed in the urban community of Cap-Haïtien. At this site, the vast majority of reported cases (87%) corresponded to patients over 5 years of age, which represents a percentage consistent with an ongoing cholera outbreak. Finally, the metropolitan area of Port-au-Prince displayed a markedly lower attack rate (1.4 cases per 10,000 inhabitants per month) than the 24 municipalities that were likely affected. Moreover, the extremely low lethality rate (0.1%) indicates that the majority of cases were probably associated with other diarrheal diseases. However, even in Port-au-Prince, a few laboratory-confirmed cases were reported at least until the beginning of March.

Currently, no evidence of persistence of toxigenic *V. cholerae* in the Haitian environment at a significant level has been reported. In fact, an environmental study performed during the warm and rainy season 2012 failed to detect toxigenic *V. cholerae* via both culture and polymerase chain reaction analyses, even in estuaries (S Baron et al., in publication process). This does not allow to completely exclude the presence of a few toxigenic *V. cholerae* bacteria in the aquatic environment; however, considering that a minimum inoculum is required to provoke cholera 14 , it is unlikely that undetectable concentrations of culturable or viable-but-non-culturable toxigenic *V. cholerae* in Haitian surface waters may greatly influence the dynamic of the current epidemic. Conversely, the remaining cases and small outbreaks that were still ongoing at the end of the dry season will thus likely play a leading role in the re-emergence of cholera during the rainy season. The fight against the infectious agent must therefore target this persisting human reservoir. As incidence of cholera decreases during the dry season, it is all the more important to enhance the fight against cholera transmission. Unfortunately, our field investigations show that low attack rates were often interpreted as evidence of “residual disease”. The few reported cases and deaths were considered as "acceptable", and the investigation of small outbreaks and the hunt for the last remaining cases were therefore neglected. Such an attitude does not appear relevant if, as stated in the governmental strategic plan to fight cholera, the aim of the struggle is to eliminate cholera 15. In 2012, the lull of the dry season had been followed by new epidemic waves responsible for 903 additional deaths 8. Such a number of deaths is too high to not consider cholera as a priority. This is all the more important now that international funding is currently lacking, which has resulted in a deterioration in the quality of care at treatment centers as recently noted in an MSF press release 13 and a halt in community prevention activities.

To enhance the effectiveness of the fight during the dry season, interventions should be targeted on active foci, which must be detected as early as possible and immediately investigated. Suspected cases should be confirmed via microbiological testing, as the risk of inaccurate cholera diagnosis is important especially when the number of reported cases diminishes. Actions should primarily focus on access to clean water via the establishment or rapid repair of distribution networks when possible and the free distribution of treatment products in the other cases. These actions are all the more effective when competent technicians apply practical solutions adapted to the local context in populations still plagued by recent cholera cases.

Vaccination should only be a supplementary element in the strategy to eliminate the disease. Indeed, a meta-analysis conducted in 2011 by the Cochrane collaboration showed that the effectiveness of current vaccines was only 52% in the first year and 62% during the second year 16. This overall rate of protection is lower in children less than 5 years of age (38% versus 66% for 5 years and older). As long-term effectiveness of the vaccine has not been demonstrated, WHO recommends re-vaccination after two years 17. Given the lack of demonstrated efficacy in young children, vaccination should therefore be reserved for individuals over 5 years of age. Vaccination programs should always be accompanied with public awareness campaigns and actions to improve access to safe water and sanitation. In the current context of vaccine shortages, massive untargeted vaccination throughout the country remains unrealistic. Instead, vaccination sites should be determined in real time based on epidemiological observations and microbiological confirmation. Inadequately targeted campaigns would have a high cost and yield poor results in impeding cholera transmission. Finally, there is a need to ensure that the financial and manpower resources required for vaccination campaigns do not impair those necessary to conduct preventive actions based on the promotion of enhancing hygienic practices and supplying safe water.

## Conclusion

As observed at the beginning of 2013, it is evident that cholera continues to affect Haiti. Epidemiological data and studies on environmental strains have shown that toxigenic *V. cholerae* O1 persists only in the human reservoir, without a significant presence in the aquatic environment. As seen in 2011 and 2012, there is a great risk that cholera will re-emerge during the upcoming rainy season. The current situation should neither be seen as an acceptable background of cases nor justify a cutback in preventive actions. On the contrary, during this period of low incidence, control activities targeting residual foci are more likely to be effective. Unfortunately, community health activities appear insufficient and poorly suited. The present analysis, mildly severe but objectively documented, should be received as an incentive to maximize efforts to prevent new outbreaks during the rainy season and ultimately eliminate cholera from Haiti and the island of Hispaniola. Not long ago, other Latin America countries were able to achieve this radical goal 18. Cholera must remain a health emergency and not a development issue. Cholera will not become endemic in the country if the disease is not endemic in the minds of the people.
